# Crosstalk between Extracellular Matrix Stiffness and ROS Drives Endometrial Repair via the HIF-1α/YAP Axis during Menstruation

**DOI:** 10.3390/cells11193162

**Published:** 2022-10-09

**Authors:** Tao Zhang, Yan Wang, Yingnan Wang, Cuiyan Liu, Chunyang Han

**Affiliations:** 1 College of Animal Science and Technology, Anhui Agricultural University, Hefei 230031, China; 2 Department of Clinical Veterinary Medicine, College of Veterinary Medicine, Huazhong Agricultural University, Wuhan 430070, China

**Keywords:** menstruation, ECM stiffness, ROS, YAP, HIF-1α

## Abstract

Although the menstrual cycle driven by sex steroid hormones is an uncomplicated physiological process, it is important for female health, fertility and regenerative biology. However, our understanding of this unique type of tissue homeostasis remains unclear. Here, we examined the biological effects of mechanical force by evaluating the changing trend of extracellular matrix (ECM) stiffness, and the results suggested that ECM stiffness was reduced and that breaking of mechanotransduction delayed endometrium repair in a mouse model of simulated menses. We constructed an ECM stiffness interference model in vitro to explain the mechanical force conduction mechanism during endometrial regeneration. We discovered that ECM stiffness increased the expression and nuclear transfer of YAP, which improved the creation of a microenvironment, in a manner that induced proliferation and angiogenesis for endometrial repair by activating YAP. In addition, we observed that physiological endometrial hypoxia occurs during the menstrual cycle and that the expression of HIF-1α was increased. Mechanistically, in addition to the classical F-actin/YAP pathway, we also found that the ROS/HIF-1α/YAP axis was involved in the transmission of mechanical signals. This study provides novel insights into the essential menstrual cycle and presents an effective, nonhormonal treatment for menstrual disorders.

## 1. Introduction

The mammalian uterus is a dynamic remodeling tissue, as evidenced by the menstrual process of higher-order primates and fast tissue remodeling without any scar after parturition. Menstruation is a routine physiological process in part of most women’s lives that is defined as a consequence of cyclical changes in hormonal activity [1,2]. However, menstrual abnormalities can lead to gynecological complications, including abnormal uterine bleeding (AUB) [1], infertility [3], menstrual pain [4], and uterine hyperplasia [5]. Regular menstrual cycles are a vital sign of women’s general health and studies have estimated that almost one-third of women’s quality of life worldwide is affected by menstrual abnormalities [6]. Despite the importance of the cycle of shedding and regeneration of the uterus during menstruation in human fertility and regenerative biology, our understanding of this unique type of tissue homeostasis remains rudimentary. 

To understand the potential mechanism of the uterus during physiological or pathological processes, previous studies have mostly focused on biochemical components, such as proteins, nucleic acids and hormones [7,8]. This is one of the main reasons why hormone drugs or surgery are routinely used clinically for the symptoms of uterine diseases. However, in the past three decades, no novel drugs for the treatment of uterine diseases have appeared, and the disadvantages of hormonal drugs have received more attention [1,9]. Therefore, exploring a new mechanism of uterine function is important in reproductive management. Although many studies have indicated that steroid hormones play an important role in the menstruation cycle, it should be noted that the endometrium stops bleeding and healing even without any hormonal support [10,11]. This has led us to speculate on the role of mechanical force in endometrial development and repair, which is involved in regulating the homeostasis of a variety of tissues and is an important part of the cell microenvironment. Mechanical forces exhibit a considerable influence on cell fate and homeostasis, with specialized molecules and structures affecting mechanosensing, and mechanotransduction occurring through signaling pathways involving biochemical signals to mediate cell behavior [12]. Previous studies suggested that the stiffness, elasticity and other mechanical properties of the endometrium change during the menstrual cycle [12,13]. For example, the stiffness of the endometrium is higher during the proliferative phase (3.34 ± 0.42 kPa) than during the early secretory phase (1.97 ± 0.34 kPa) in women [14]. However, past work has rarely addressed the mechanism of mechanical cues in the uterus.

YAP (also known as YAP1) and the highly related transcriptional regulator TAZ (also known as WWTR1) are conserved sensors of cell mechanics that sense a very diverse set of mechanical cues, such as extracellular matrix (ECM) rigidity, cell geometry and flow forces and translate them into cell-specific transcriptional programs [15,16]. YAP/TAZ activity is indispensable for normal homeostasis in several adult organs, and abnormal YAP/TAZ activation causes multiple diseases, including fibrosis and cancer, and is critical for regeneration upon injury [17,18]. The Hippo cascade is a prime input for YAP/TAZ activity and the first mechanism discovered for YAP/TAZ regulation. In fact, YAP/TAZ was initially observed in mutants of the Hippo cascade, which is a prime input for YAP/TAZ activity through phosphorylation by kinases [19]. However, a wealth of recent evidence indicates that the YAP/TAZ response to cellular mechanotransduction can operate in a LATS-independent manner, which may depend on diverse biomechanical signals, types of cells and mechanical stresses [20,21]. Previous studies have shown that the biomechanical cues of ECM stiffness and cell adhesion are involved in the maintenance of early pregnancy [18], but the mechanism of mechanotransduction in the endometrium has not been clarified.

In this study, we hypothesized that the change in mechanical force in the endometrium during menstruation may create a special microenvironment for endometrial regeneration. Thus, we established an experimental model using mice with simulated menses and endometrial stromal cells (ESCs) and assessed the repair process of the endometrium after mechanical force interference. 

## 2. Materials and Methods

### 2.1. Mice

Kunming (KM) female (aged 6–8 weeks) mice were purchased from the Laboratory Animal Service Centre of Anhui Agricultural University. All mice in this study, unless otherwise indicated, were nonpregnant female mice at the proestrus stage. All animal experiments were approved by the Animal Experiment Committee of Anhui Agricultural University and conducted in accordance with the university guidelines on animal experimentation. 

### 2.2. Mouse Model of Menstruation and Treatment Regimens

**Figure 1:**
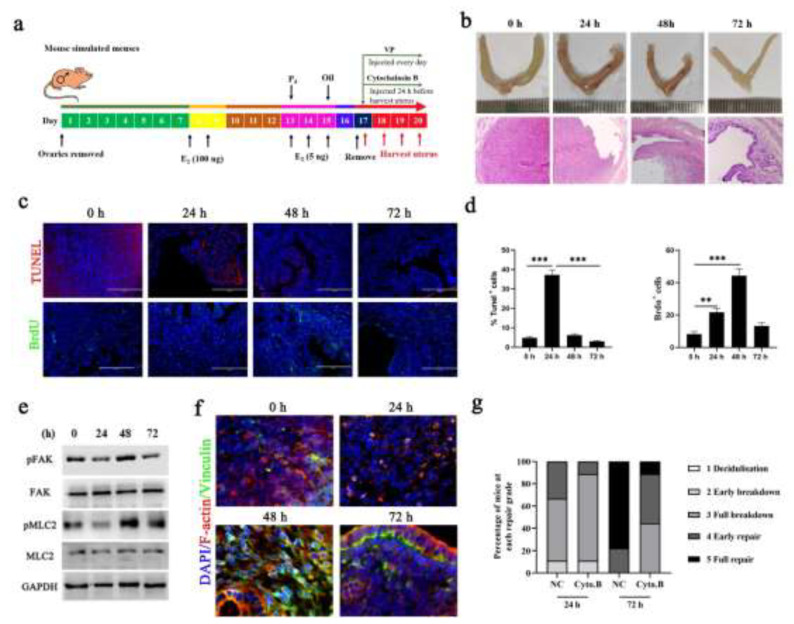
**ECM stiffening features of the endometrium in a mouse model of simulated menses**. (**a**) Diagram depicting the murine model of simulated menstruation. E2 17-β-estradiol, P4 progesterone. Timed to 0 h, 24 h, 48 h and 72 h based on P4 withdrawal time. (**b**) Representative whole uterus images (top) and histopathological images of paraffin-embedded mouse uterine sections (bottom, n = 2). Scale bar: 400 μm. (**c**,**d**) Representative images of uterine sections stained with TUNEL (top, n = 2) and BrdU (bottom, n = 2) and quantification of TUNEL+ and BrdU+ positive cells ((**d**), n = 5). Scale bars, 200 μm. The data are presented as mean ± SEM. ** *p* < 0.01; *** *p* < 0.001. (**e**) Western blot analysis of pFAK, FAK, pPMLC2 and pMLC2 expression. GAPDH was used as a loading control. (**f**) Representative images of vinculin and F-actin in mouse uterine sections. Scale bar, 20 μm. n = 4. (**g**) Endometrial histological breakdown/repair score at 24 h and 72 h in mice treated with DMSO and Cyto. B. Graphs represent the percentage of mice at each histological grade per experimental group. n = 9. Experiments were repeated n times with duplicate biological replicates.

As previously described, a mouse model of menstruation was simulated in ovariectomized mice (Figure 1a) [5,22]. All surgeries were performed under isoflurane-induced anesthesia. In brief, one week after ovariectomy mice received s.c. injections of 100 ng of 17-β-estradiol (E2, Sigma) in ethanol/arachis oil (1:9) on three consecutive days. After 3 days of rest, progesterone (P4, Sigma) implants were inserted s.c. into the back of each mouse on Day 13. The mice also received daily injections of 5 ng E2 in Arachis oil from Days 13 to 15. On Day 15, 20 μL of sesame oil was injected into the lumen of the right uterine horn of each mouse to induce decidualization. The left horn remained untreated as a control. P4 withdrawal was induced 2 days after decidualization (Day 17) by removal of the P4 implant. Mice were killed 12 h, 24 h, 36 h, 48 h and 72 h thereafter, and the uteri were harvested for further analysis. DMSO (1%; Sigma) and verteporfin (VP, 10 μmol/kg, Macklin Biochemical) were intraperitoneally injected into the mice every day after P4 withdrawal. Cytochalasin B (Cyto. B, 50 mg/kg, AbMole) was intraperitoneally injected into the mice 24 h before uterine collection. All mice received an intraperitoneal injection of 100 μL bromodeoxyuridine (BrdU, 75 mg/kg, Sigma-Aldrich) 12 h prior to culling.

### 2.3. Primary Culture and Treatment

Mouse endometrial stromal cells (ESCs) were isolated and cultured as previously described [18]. In brief, uterine horns were harvested from pregnant mice on Day 5 and finely chopped using scalpel blades. The minced tissue was then incubated in PBS sterile digestive solution containing 1% collagenase II (Sigma-Aldrich) at 37 °C for 1 h. Tissue in chelation buffer was filtered through a 40 μm filter mesh to remove undigested material and centrifuged at 1000 r/mins for 5 min, followed by 3 additional washes in washing medium. The digested suspension was centrifuged, and the cells were resuspended in DMEM containing 15% FBS (Gibco), 50 IU/mL penicillin, 50 IU/mL streptomycin, and 2.5 μg/mL amphotericin B (all from HyClone). Endometrial epithelial and stromal cell populations were isolated by differential adherence. After 4 passages, cell purity was detected by immunofluorescence staining for the endometrial epithelial cell marker CK18 and the stromal cell marker vimentin. Note that primary cells were used for the next experiment after four passages. Meanwhile, ESCs were treated with 10 nM E2 plus 1 μM P4 in DMEM-F12 containing 2% charcoal-treated FBS (Gibco) for in vitro decidualization, which was verified as previously described [23,24]. 

Based on previous studies [18], we constructed a gradient model of extracellular matrix stiffness in 2D culture. Briefly, hydrogels of different hardness were formulated using the relevant reagents. Hydrogels with hardnesses of 1 kPa and 40 kPa were configured in this study. Then, depending on the size of the hydrogel and the cell needs, the hydrogel was coated overnight with 10 μg/mL bovine fibrinogen (Solarbio, Beijing, China) before using the photosensitizer Sulfo-SANPAH (Thermo Fisher Scientific, USA). Activation of the hydrogel requires irradiation with UV light at 565 nm, and after aseptic processing, the hydrogel can be used for cell culture. Transient transfection of cells was carried out using Lipofectamine 2000 (Invitrogen, CA, USA) as recommended by the manufacturer. Briefly, si-NC, si-YAP, si-HIF-1α and pcDNA 3.1(+) YAP were each combined with Lipofectamine 2000 in serum-free Opti-MEMI medium (Gibco) for 5 min, and then the two were mixed and incubated for 20 min at room temperature. The mixture was added to cells cultured in a 6-well plate and the complete medium was replaced after 6 h of culture. All RNAi oligonucleotides and plasmids were obtained from GeneCreate (Wuhan, China). 

### 2.4. Immunofluorescence 

Cells grown on glass coverslips were fixed with 4% PFA for 15 min at room temperature (RT), washed 3 times with PBS and then permeabilized with 0.05% Triton X-100 for 10 min at RT. Paraffin-embedded slides were dewaxed and rehydrated. Tissue sections were permeabilized with PBS containing 0.2% Triton X-100 before blocking with 10% normal goat serum for 1 h at RT. After washing 3 times with PBS, the sections were stained with primary antibodies in humidified chambers at 4 °C overnight. After extensive washing with PBS, the sections were incubated with fluorescent secondary antibodies for 2 h at RT in the dark and phalloidin (depending on the staining) for 4 h at RT. DAPI staining was performed after extensive washing. Sections were observed and photographed using a Nikon Eclipse C1 imager (Japan), a Nikon DS-U3 imager (Carl Zeiss) or a light microscope (Olympus, Japan). All antibodies (references and dilutions) are presented in Table S1. For quantification of YAP/TAZ subcellular localizations, BrdU- or YAP-positive cells were counted in at least 9 fields of view using ImageJ software, and at least 2 samples per experimental group were analyzed. 

### 2.5. Measurement of Intracellular ROS Generation

As described previously, DCFH-DA (Sigma, MO, USA) was used to measure ROS production. A flow cytometer (BD Biosciences, San Jose, CA, USA) was used to measure DCF fluorescence, and FlowJo10 was used to analyze the data.

### 2.6. Cell Cycle Analysis

After treatment with siYAP and pcDNA3.1(+) YAP in vitro decidualized cells were fixed with 70% ice-cold alcohol and stained with PI/RNase stained buffer (BD Biosciences). Finally, the cells were analyzed by flow cytometry.

### 2.7. Total Antioxidant Ability and Antioxidant Enzyme Activity in the Uterus

The total antioxidant capacity of uterine samples was tested using a Total Antioxidant Capacity (T-AOC) Assay Kit (Beyotime, China Shanghai) with the FRAP (ferric reducing ability of plasma) method. SOD activity (Beyotime Biotechnology, Shanghai, China) was determined using commercial kits following the product manuals, as previously described [23].

### 2.8. qRT-PCR

Freshly isolated tissue or cultured cells were lysed in TRIzol reagent (Invitrogen, Carlsbad, CA, USA) and cDNA was synthesized using the HiScript^®^ II Q Select RT SuperMix from a qPCR kit (Vazyme Biotech Co., Ltd., Nanjing, China). Quantitative RT–qPCR was performed in duplicate using FastStart Universal SYBR Green Master Mix (Roche Applied Science, Mannheim, Germany) and the StepOne real-time PCR System (Life Technologies Corp., Waltham, MA, USA). Expression levels are given relative to GAPDH. The sequences of the primers are provided in Table S2.

### 2.9. Western Blots

Total protein from cells or tissue was extracted using a protein extraction kit (Vazyme, Nanjing, China). Nuclear and cytoplasmic proteins were extracted according to the instructions of the Nuclear and Cytoplasmic Protein Extraction Kit (Sangon Biotech, Shanghai, China). The protein was separated by SDS–PAGE (5–12%), transferred onto polyvinylidene difluoride membranes (Solarbio, Beijing, China) and blocked in 10% nonfat milk in TBST for 2 h at RT. The membranes were successively incubated with primary antibodies overnight at 4 °C. Following three washes with TBST, the membranes were then incubated with secondary antibodies at RT for 2 h. After three washes, the membranes were subjected to chemiluminescence using Clarity Western ECL Substrate (Affinity, Changzhou, China). β-actin and Lamin B1 were used as standard proteins for cytoplasmic and nuclear proteins, respectively. Protein expression was detected using an enhanced chemiluminescence detection system (ImageQuant LAS 4000 mini, USA). Antibody information is provided in Table S1.

### 2.10. Histological Analysis

The uterus was fixed in 4% paraformaldehyde (PFA) solution and the tissue was embedded in paraffin. Five micrometer mouse uterine sections were stained with hematoxylin and eosin (H&E), and the stage of injury/repair was graded by two masked independent observers using a previously published scoring system [25,26]. 

### 2.11. TUNEL Assay

Apoptosis of endometrial tissue cells was identified by TUNEL staining and the specific operation method was performed according to the manufacturer’s instructions (Roche Diagnostics GmbH) as described previously [27]. The number of TUNEL+ positive cells was counted in at least 5 fields of view in one individual mouse and at least 2 mice per experimental group were analyzed.

### 2.12. Statistics and Reproducibility 

As shown in the figure legends, all experimental repetitions in the text are indicated by n values, which represent n independent experiments or samples, and all quantitative data are expressed as the mean ± SD or the mean ± S.E.M. Statistical analysis was performed using GraphPad Prism 8. Statistical significance was tested using the unpaired Student’s *t*-test or one-/two-way ANOVA and Sidak’s multiple comparison test. All data were considered statistically significant at * *p* < 0.05 and ** *p* < 0.01.

## 3. Results

### 3.1. Mechanotransduction Signaling of ECM Stiffness Is Involved in Mouse Menstrual Progression

To assess and manipulate mechanical force we used a mouse model of endometrial damage and regeneration that simulates menstruation according to previous studies [5,22] (Figure 1a). We first evaluated the normal pattern of pathological characteristics and the repair time in the menstrual uterus. The complete menstruation-like breakdown was observed at 24 h after the withdrawal of P4, followed by active repair and complete regeneration by 72 h (Figure 1b). This is supported by the level of apoptosis and proliferation of endometrial cells (Figure 1c,d). Interestingly, the above results are slightly different from those of previous studies [5,10], which may depend on the methods of evaluating repair, the type of mice and the subjective interpretation of uterine sections. 

Previous studies have confirmed that the ECM stiffness of the endometrium decreases during the menstrual cycle [12,14]. Consistent with this phenomenon, we found that the phosphorylation levels of focal adhesion kinase (FAK) and myosin light chain 2 (MLC2), which are ECM receptors and function as mechanotransducers, were increased during the proliferative phase (Figure 1e). Staining with vinculin, a marker of adherens junctions, showed that vinculin expression decreased in menstrual cells (Figure 1f). We also observed that the actin cytoskeleton was involved in mechanotransduction during the uterine menstruation cycle. Next, we inhibited F-actin contractility by injection with inhibitors of F-actin cytochalasin B after the withdrawal of P4 and the results suggested that the repair period of the mouse uterus was significantly prolonged (Figure 1g). These results indicate that ECM stiffness enables timely repair of the denuded endometrial surface during menstruation. 

### 3.2. Inhibition of YAP Activation Prevents Efficient Repair

**Figure 2:**
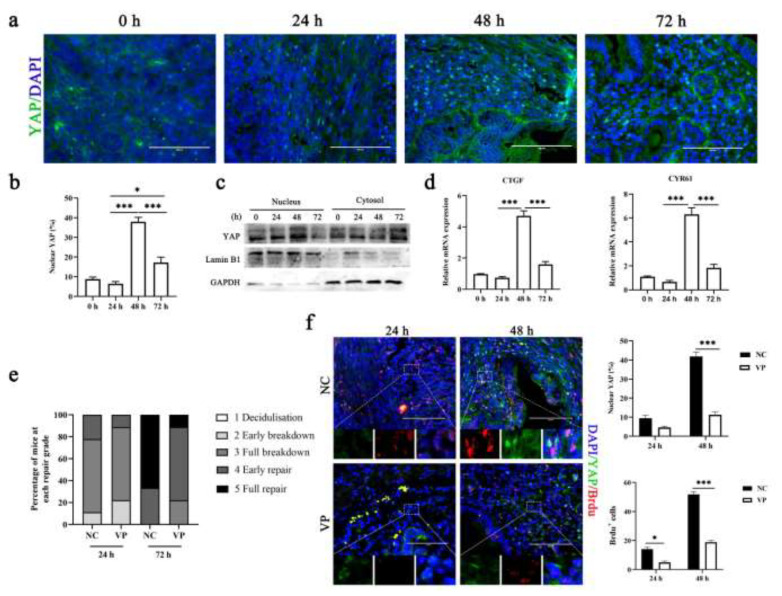
**Pharmacological inhibition of YAP at menses delays repair**. (**a**,**b**) Representative immunofluorescence images ((**a**), n = 2) and quantification of nuclear and cytoplasmic subcellular localization ((**b**), n = 9) of YAP in uterine sections. Scale bar: 100 μm. (**c**) Western blotting for YAP in nuclear and cytoplasmic protein fractions from mouse endometrial tissue. n = 3. Lamin B1 and GAPDH were used as loading controls for nuclear and cytoplasmic proteins, respectively. (**d**) RT-qPCR analysis of CYR61 and CTGF mRNA levels. n = 3. (**e**) Endometrial histological breakdown/repair score at 24 h and 72 h in mice treated with DMSO and VP. The graphs represent the percentage of mice at each histological grade per experimental group. (**f**) Representative immunofluorescence images (left, n = 2) and quantification of YAP- and BrdU-the positive cells ((**f**), n = 5) in the uterus of mice uterine treated with DMSO or VP at 24 h and 48 h. Scale bar: 100 μm. Enlarge: 10 μm. The data are presented as the mean ± SEM. Experiments were repeated n times with duplicate biological replicates. * *p* < 0.05, *** *p* < 0.001.

To understand the mechanism of ECM stiffness changes during the menstruation cycle, we assessed the activity of YAP, which is a classical sensor of mechanical force instructed by the cellular microenvironment. We observed that the levels of YAP were significantly elevated and translocated to the nucleus from the menstrual phase to the proliferative phase (Figure 2a,b). Consistent with those results, the mRNA expression levels of CYR61 and CTGF mRNAs, two well-known Hippo-YAP target genes, were increased in the proliferative phase (Figure 2c). To verify that activated YAP expression regulated endometrial regeneration, we administered verteporfin (VP) to mice to disrupt YAP nuclear activity. As expected, inhibition of YAP activation prolonged the repair time, characterized by decreased uterine section scores (Figure 2d). This is due to a reduction in the rate of proliferation of endometrial cells (Figure 2e). Altogether, these findings suggest that the activity of YAP mediates endometrial repair following menstruation.

### 3.3. Hypoxia Can Activate YAP by Increasing HIF-1α Expression

**Figure 3:**
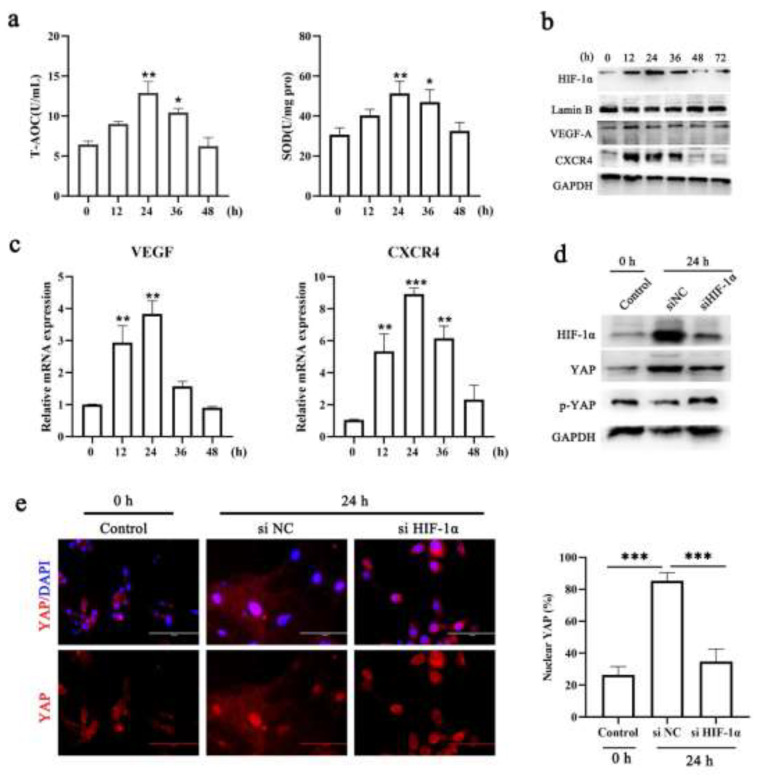
**Hypoxia sustains HIF-1α protein stability to activate YAP**. (**a**) Analysis of the antioxidant indices T-AOC activity and SOD activity in endometrial tissue. n =3. (**b**) The protein levels of VEGF, CXCR4, and HIF-1α were evaluated in the uterus by Western blotting. n = 3. (**c**) Comparison of the mRNA expression levels of VEGF and CXCR4 in isolated mouse endometrial tissue during menstruation. n = 3. (**d**) Lysates of ESCs transfected with siYAP and siNC after withdrawing P4 were analyzed for the presence of the indicated proteins. n = 3. (**e**) Confocal immunofluorescence images (left, n = 2) and quantification of nuclear and cytoplasmic subcellular localization (right, n = 15) of YAP in decidualized cells transfected with siYAP. Scale bar: 100 μm. The data are presented as the mean ± SEM. Experiments were repeated n times with duplicate biological replicates. * *p* < 0.05, ** *p* < 0.01; *** *p* < 0.001.

Cellular homeostasis is characterized by a baseline level of reactive oxygen species (ROS), which are key molecules that participate in a dynamic relationship within the endometrium [28]. However, the presence and role of hypoxia in the menstrual cycle remain controversial [29,30]. Here, we found that activities of total antioxidant capacity (T-AOC) and superoxide dismutase (SOD) were increased in the menses and early proliferation phases, indicating transient hypoxia in the uterus during menstruation (Figure 3a). Hypoxia-inducible transcription factor-α (HIF-1α) is the master and first regulator of the cellular response to hypoxia, and we found that the nuclear protein level of HIF-1α was increased in the menses and early proliferation phases (Figure 3b). Next, we found that the mRNA levels of VEGF and CXCR4, which are targets of HIF-1α that have putative roles in endometrial repair [25,31], were increased in the menses phase and that the protein level was slightly increased at 24 h following P4 withdrawal (Figure 3c). These results are consistent with a number of previous studies suggesting that HIF-response elements (e.g., CXCR4, CXCL12, VEGF) levels are significantly up-regulated at menstruation and have putative roles in endometrial repair and regeneration in the human endometrium [31,32,33]. Under in vitro decidualization, HIF-1αinduces YAP activation by inhibiting phosphorylation (Figure 3d,e). It should be noted that there is no significant activation of YAP during menstruation in vivo, implying that the presence of other signals that regulate YAP, such as ECM stiffness, predominate. Previous studies have demonstrated that hypoxic conditions are unnecessary for menstrual endometrium breakdown [34], but HIF-1α is involved in the repair of the endometrium [25]. Therefore, we speculated that HIF-1α is involved in uterine repair during the menses phase in a mechanical force-dependent manner.

### 3.4. ECM Stiffness Mediated the Activation of HIF-1α and ROS Production by Regulating YAP Activity

**Figure 4:**
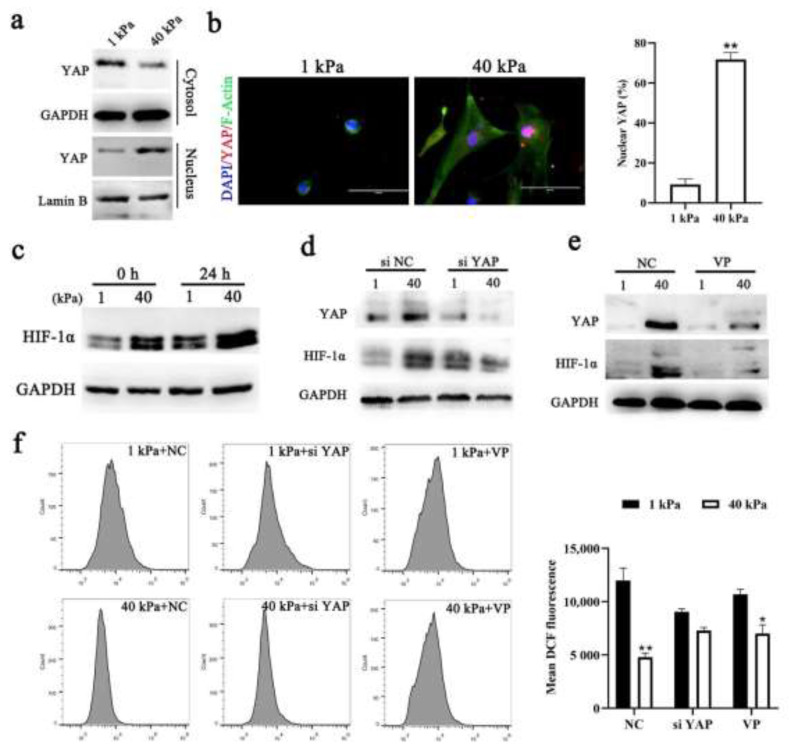
**ECM stiffness regulates the activity of YAP/HIF-1α and ROS production in ESCs**. (**a**) Western blotting for YAP in nuclear and cytoplasmic protein fractions from ESCs plated on 1 kPa or 40 kPa fibronectin-coated hydrogels for 24 h, n = 3. (**b**) Confocal immunofluorescence images (left, n = 2) and quantification of nuclear and cytoplasmic subcellular localization (right, n = 15) of YAP in ESCs plated on hydrogels with different rigidities. Scale bar, 10 μm. (**c**) Western blotting for HIF-1α from decidualized cells plated on 1 kPa or 40 kPa hydrogels for 0 h and 24 h, n = 3. (**d**,**e**) Western blot analysis of HIF-1α and YAP expression in decidualized cells treated with siYAP (**d**) or VP (**e**) plated on 1 kPa or 40 kPa hydrogels for 24 h, n =3. (**f**) The intracellular ROS levels were measured by staining with DCFH-DA and then determined by flow cytometry. n = 2. The data are presented as the mean ± SEM. Experiments were repeated n times with duplicate biological replicates. * *p* < 0.05 and ** *p* < 0.01.

To determine whether the regulation of endometrial repair by ECM stiffness is mediated by ROS, we constructed a hydrogel culture model of endometrial stromal cells (ESCs) in vitro. Similar to our previous study [18], the level of YAP was increased and entered the nucleus in ESCs grown in stiffness hydrogels (~40 kPa) (Figure 4a,b). Unsurprisingly, HIF-1α showed the same expression pattern as in decidualized cells (Figure 4c). Next, we blocked mechanotransduction by knocking down YAP or using VP, an inhibitor of YAP, and examined the effects (Figure 4d). The expression level and subcellular localization of HIF-1α regulated by ECM stiffness were abolished (Figure 4d,e). This shows that YAP is upstream of HIF-1α. Interestingly, we noted that ECM stiffness could reduce extracellular ROS levels by activating YAP (Figure 4g). These results suggest the presence of crosstalk between ECM stiffness and ROS under physiological conditions.

### 3.5. YAP Activation Is Required for Endometrial Regeneration during Menstruation

**Figure 5:**
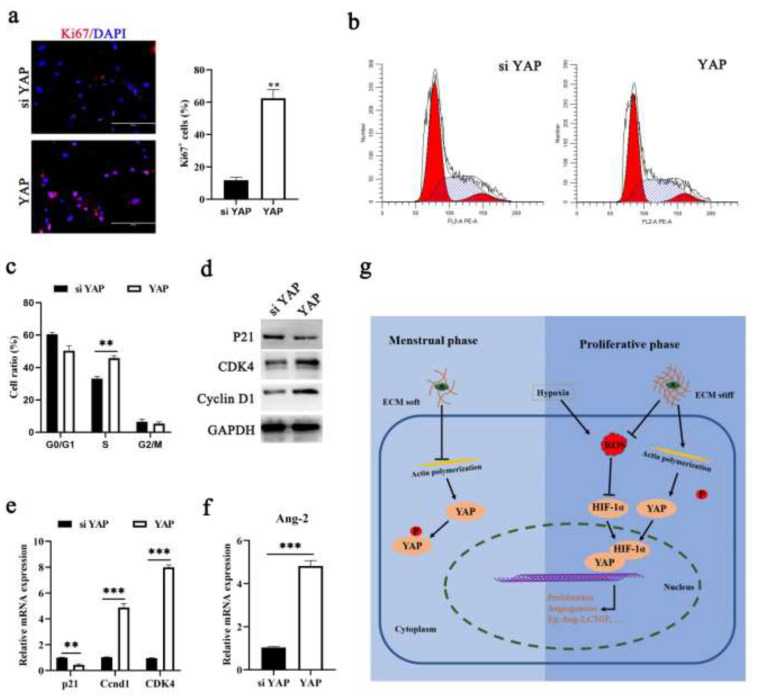
**YAP activation provides the physiological environment needed for endometrial repair**. (**a**) Confocal immunofluorescence images (**left** n = 2) and quantification of Ki67 positive cells (**right**, n = 9) in ESCs transfected with siYAP and pcDNA 3.1 (+) YAP. Scale bars, 200 μm. (**b**,**c**) Schematic diagram of cell cycle detection results. n = 3. (**d**,**e**) Protein and mRNA expression levels of p21, cyclin D1 and CDK4 were detected in ESCs transfected with si YAP or pcDNA 3.1 (+) YAP, n = 3. (**f**) RT-qPCR analysis of Ang-2 expression levels in ESCs transfected with siYAP or pcDNA3.1(+) YAP, n = 3. The data are presented as the mean ± SEM. Experiments were repeated n times with duplicate biological replicates. ** *p* < 0.01, *** *p* < 0.001. (**g**) Schematic images depicting upregulation of ECM stiffness and ROS upon nuclear translocation and activation of YAP/HIF-1α and subsequent binding to TEAD, Ang2, and CTGFdriving endometrium repair after in the menstrual cycle.

To determine the impact of YAP on endometrial repair, we used siYAP or pcDNA 3.1(+) YAP to induce the expression of YAP in ESCs. The results of proliferation analysis showed that the activation of YAP promoted cell proliferation (Figure 5a,b). In our assessment of cyclin proteins, we also found that YAP induced high expression of cyclin D1 and cyclin-dependent kinase 4 (Cdk4), which are principal regulators in the G1/S phase transition (Figure 5c–e). Angiogenesis plays a key role in shedding and repair during the menstrual cycle [1]. Ang-2 and VEGF regulate angiogenesis in a pro- or antiangiogenic manner, depending on the cell context, which is an autocrine factor. Here, we confirmed that YAP binding HIF-1α induces the secretion of VEGF during the proliferative phase, and speculated that YAP might regulate Ang-2. We found that higher Ang-2 expression was detected in the presence of ECM stiffness and in ESCs with YAP overexpressed (Figure 5f). These results together suggest that ECM stiffness and ROS are critically involved in endometrial regeneration by regulating YAP activity. 

## 4. Discussion

As a routine part of life, the characteristics of the menstrual cycle are considered additional vital signs in assessing women’s general health status [6]. Menstrual abnormalities are associated with many common diseases, including cancer, endometriosis, heavy menstrual bleeding (HMB) and infertility, that collectively influence a woman’s quality of life and mental health problems [1,8]. Irregular and excessive menstruation has become increasingly prominent in women’s health for nearly half a century, probably due to increased awareness and prevalence [35]. Thus, dissecting the mechanisms that regulate endometrial repair across the menstrual cycle is crucial for developing novel treatment strategies for menstrual disorders. Here, we reveal that ECM stiffness was reduced and breaking of mechanotransduction decreased the activity of YAP and delayed endometrium repair in a mouse model of simulated menses. Furthermore, our discovery indicates that ECM stiffness activates YAP not only through the classical mechanotransduction pathway but also by affecting ROS levels and HIF-1a activity, thereby improving the microenvironment for endometrial repair (Figure 5g).

The expansion of the mechanobiology field over the past decade has advanced our understanding of the effects on cellular behavior of sensing and responding to mechanical forces [36]. It is becoming increasingly clear that mechanical forces contribute to development, homeostasis and regeneration after damage [37,38]. Previous studies have rarely addressed the role of cellular mechanics in the menstrual cycle. In fact, almost all biological processes are subject to and generate forces, which can vary depending on the context [37,39]. Three-dimensional multifrequency magnetic resonance elastography and ultrasound elastography can provide mechanical parameter maps of the uterus, and prior results that indicated the stiffness of the endometrium decreases during the menstrual cycle [14]. In this study, we revealed that ECM stiffness-mediated mechanotransduction pathways are activated during the proliferative phase in a mouse model of simulated menses. We administered cytochalasin B to block mechanical signaling at the time of simulated menstruation in a mouse model, which significantly prolonged endometrial repair time. Our previous study also showed that ECM stiffness and cell adhesion were altered in the endometrium during pregnancy [18]. However, the mechanism of uterine function regulation, such as menstruation mediated by mechanical cues, is still unclear.

Cells have multiple pathways for sensing and responding to extracellular mechanical forces, such as ion channels and mechanosensitive proteins [38]. Mechanotransduction of ECM stiffness is often translated into biochemical signaling cascades through protein deformation caused by cell-generated forces loading on mechanosensitive proteins [36,40]. YAP and TAZ are paralogous proteins that act as major downstream targets of the Hippo pathway and sense biochemical and mechanical cues in the tissue environment to communicate with it [16]. Recently, we and other groups have revealed that YAP/TAZ activity is regulated by ECM stiffness and cell density [16,18]. Here, we found that YAP was activated by ECM stiffness during the proliferative phase of the menstrual cycle. Knockdown or inhibition of YAP activation and nuclear retention significantly prolonged the menstrual repair period. Similarly, our studies indicated that the conformation and tension of the F-actin cytoskeleton mediated the regulation of YAP by ECM stiffness. YAP can potentially contribute to many transcription factor signaling pathways, including the TEAD1-4, STAT3 and CTGF pathways, by acting as a partner to regulate cell behavior [15,16,41]. Our results suggest that YAP induced cell proliferation and acceleration of the G1/S phase transition in mouse models of simulated menses and ESCs, which is consistent with prior findings in endometrial epithelial cells and other types of cells within and outside our department [18,42]. 

Herein, we detected the level of extracellular ROS and found hypoxia during the menstrual and early proliferative phases in a mouse model of simulated menses. In fact, the level and role of ROS during the menstrual cycle have remained the subject of intense controversy, with conflicting results from different laboratories utilizing in vitro, uterus explant and in vivo models [25,29,34]. The consensus is that the withdrawal of progesterone leads to strong vasoconstriction of the endometrial spiral arterioles and hypoxia, but hypoxia is not necessary for the shedding of endometrial tissue [8,43]. Previous studies have revealed that HIF-1α regulated the expression of hypoxia-regulated repair factors (VEGF, CXCR4 and ADM), and then accelerates uterine repair via stimulation of angiogenesis and promotes the survival of ESCs after menstruation [44]. It should be noted that the regulation of HIF-1α activity is not completely dependent on the hypoxic environment [25,45]. Here, we constructed a model of induced in vitro decidualization of stromal cells and found that HIF-1α expression increased after simulated progesterone withdrawal, and this increase affected YAP activation. We also confirmed that ECM stiffness regulates the activity of YAP dependent on the polymerization of F-actin in ESCs. In an in vitro decidualization model, we found that ECM stiffness regulated HIF-1α stabilization by modulating YAP activity after the withdrawal of hormones. Previous studies have shown that mechanical force can regulate extracellular ROS levels and is affected by cell type and force sensitivity thresholds [46,47]. Our results suggest that ECM stiffness is involved in the regulation of extracellular ROS levels by regulating YAP activity. It is not difficult to conclude the presence of a feedback mechanism by which ECM stiffness and hypoxia crosstalk affect endometrial repair.

In summary, we showed that an increase in ECM stiffness during the early proliferative phase promotes uterine repair by inducing YAP activation. Mechanistically, in addition to the classical F-actin/YAP pathway, we also found that the ROS/HIF-1α/YAP axis is involved in the transmission of mechanical signals. Our results suggest an essential role of ECM stiffness in menstrual physiology thus indicating a novel therapeutic target for irregular and long menstrual-induced uterine disorders.

## Supplementary Materials

The following supporting information can be downloaded at: https://www.mdpi.com/article/10.3390/cells11193162/s1, Table S1: Information on antibodies used in the study; Table S2: oligonucleotide primers used for qPCR.
