# Supplementary material for: Crosstalk between Extracellular Matrix Stiffness and ROS Drives Endometrial Repair via the HIF-1α/YAP Axis during Menstruation
Source: Cells. 2022 Oct 9;11(19):3162. doi: 10.3390/cells11193162 (PMC9562179; doi:10.3390/cells11193162)
Supplement: Supplementary file 1 [file cells-11-03162-s001.zip › cells-1916613-supplementary.pdf]

Crosstalk between ECM stiffness and ROS drives endometrial repair via HIF-1α/YAP axis during menstruation

Tao Zhang*[1,2], Yan Wang[1], Cuiyan Liu[1], Chunyang Han[1]

[1] College of Animal Science and Technology, Anhui Agricultural University, Hefei 230031, People's Republic of China.

[2] Department of Clinical Veterinary Medicine, College of Veterinary Medicine, Huazhong Agricultural University, Wuhan 430070, People's Republic of China.

*** Correspondence**

Tao Zhang, College of Animal Science and Technology, Anhui Agricultural University, Hefei 230031, People's Republic of China.

E-mail: taozhang@ahau.edu.cn

Contents

Supplement Table S1- S2

Table S1. Information on antibodies used in the study.

Antibodies	Product code	Dilution	Species	Manufacturer
GAPDH	#5174	1:1000 (WB)	Rabbit	Cell Signaling Technology
Anti-rabbit IgG	#5127	1:2000 (WB)	mouse	Cell Signaling Technology
Anti-mouse IgG	#7056	1:2000 (WB)	Goat	Cell Signaling Technology
YAP	#14074	1:1000 (WB), 1:250 (IF)	Rabbit	Cell Signaling Technology
pYAP	#13008	1:1000 (WB)	Rabbit	Cell Signaling Technology
FAK	#3285	1:1000 (WB)	Rabbit	Cell Signaling Technology
pFAK	#3281	1:1000 (WB)	Rabbit	Cell Signaling Technology
pMLC2	#3671	1:1000 (WB)	Rabbit	Cell Signaling Technology
MLC2	#3672	1:1000 (WB)	Rabbit	Cell Signaling Technology
HIF-1α	#36169	1:1000 (WB)	Rabbit	Cell Signaling Technology
Vinculin	A14193	1:50 (IF)	Rabbit	Abclonal
Brdu	A1482	1:200(IF)	mouse	Abclonal
Ki67	ab15580	1:100 (IF)	Rabbit	abcam
Lamin B1	ab133741	1:5000(WB)	Rabbit	abcam
VEGF-A	AF5131	1:1000 (WB)	Rabbit	Affinity
CXCR4	AF5279	1:1000 (WB)	Rabbit	Affinity
P21	AF6290	1:1000 (WB)	Rabbit	Affinity
CDK4	DF6102	1:1000 (WB)	Rabbit	Affinity
Cyclin D1	AF0931	1:1000 (WB)	Rabbit	Affinity
Myosin IIC	DF4196	1:500 (WB)	mouse	Affinity
YAP	sc-376830	1:1000 (WB), 1:50 (IF)	mouse	Santa Cruz Biotechnology
Cy3 anti-rabbit IgG	GB21303	1:100 (IF)	Goat	Servicebio
Cy3 anti-mouse IgG	GB21301	1:100 (IF)	Goat	Servicebio
FITC anti-mouse IgG	GB22301	1:50 (IF)	Goat	Servicebio
FITC anti-rabbit IgG	GB22303	1:50 (IF)	Goat	Servicebio
FITC Phalloidin	G1028	1:200 (IF)		Servicebio
TRITC Phalloidin	G1041	1:200 (IF)		Servicebio

Table S2 oligonucleotide primers used for qPCR.

Gene	Forward	Reverse
YAP	GCGGTTGAAACAACAGGAATTA	TGAGACATCCCAGGAGAAGA
VEGF	ACATTGGCTCACTTCCAGAAACAC	GGTTGGAACCGGCATCTTTATC
Ang-2	TCCAAGAGCTCGGTTGCTAT	CCATCTTCTCGGTGTTGGAT
CXCR4	TGGAACCGATCAGTGTGAGT	GGCAGGAAGATCCTATTGA
CDK4	ATGGCTGCCACTCGATATGAA	TCCTCCATTAGGAACTCTCACAC
Cyclin D1	GCGTACCCTGACACCAATCTC	CTCCTCTTCGCACTTCTGCTC
p21	CCTGGTGATGTCCGACCTG	CCATGAGCGCATCGCAATC
CYR61	AGCCTCGCATCCTATACAACC	TTCTTTCACAAGGCGGCACTC
CTGF	CCAATGACAACGCCTCCTG	TGGTGCAGCCAGAAAGCTC
GAPDH	CCATGTTCGTCATGGGTGTG	CAGGGGTGCTAAGCAGTTGG
